# Regulation of LRRK2 promoter activity and gene expression by Sp1

**DOI:** 10.1186/s13041-016-0215-5

**Published:** 2016-03-22

**Authors:** Juelu Wang, Weihong Song

**Affiliations:** Department of Psychiatry, Townsend Family Laboratories, Graduate Program in Neuroscience, The University of British Columbia, 2255 Wesbrook Mall, Vancouver, BC V6T 1Z3 Canada

**Keywords:** LRRK2, Sp1, Parkinson’s disease, Gene regulation

## Abstract

**Background:**

The dopaminergic neurodegeneration in the nigrostriatal pathway is a prominent neuropathological feature of Parkinson’s disease (PD). Mutations in various genes have been linked to familial PD, and leucine-rich repeat kinase 2 (LRRK2) gene is one of them. LRRK2 is a large complex protein, belonging to the ROCO family of proteins. Recent studies suggest that the level of LRRK2 protein is one of the contributing factors to PD pathogenesis. However, it remains elusive how LRRK2 is regulated at the transcriptional and translational level.

**Results:**

In this study, we cloned a 1738 bp 5’-flanking region of the human LRRK2 gene. The transcriptional start site (TSS) was located to 135 bp upstream of translational start site and the fragment −118 to +133 bp had the minimum promoter activity required for transcription. There were two functional Sp1- responsive elements on the human LRRK2 gene promoter revealed by electrophoretic mobility shift assay (EMSA). Sp1 overexpression promoted LRRK2 transcription and translation in the cellular model. On the contrary, application of mithramycin A inhibited LRRK2 transcriptional and translational activities.

**Conclusion:**

This is the first study indicating that Sp1 signaling plays an important role in the regulation of human LRRK2 gene expression. It suggests that controlling LRRK2 level by manipulating Sp1 signaling may be beneficial to attenuate PD-related neuropathology.

## Background

Parkinson’s disease (PD) is the second most common neurodegenerative disorder, affecting 1–2 % of individuals older than 65 years of age and 4–5 % of people who are 85-year-old [1–3]. Its clinical manifestations are characterized by bradykinesia, resting tremor, muscular rigidity and postural instability [4]. Pathologically, there are two prominent features seen in PD patients. One is severe and relatively selective dopaminergic neurodegeneration in the nigrostriatal pathway, which underlies the deficits in the motor systems [5, 6]. The other is cytoplasmic Lewy bodies (LBs), which primarily consist of aggregated alpha-synuclein [7]. Although tremendous efforts have been put into discovering the effective therapies, most of the treatments today are only palliative instead of modifying disease progression. Over the past years, several genes with mutations have been identified in the familial PD cases, including alpha-synuclein (SNCA) [8, 9], leucine-rich repeat kinase 2 (LRRK2) [2, 3], Parkin [10], PTEN induced putative kinase 1 (PINK1) [11], DJ-1 [12], ATP13A2 or PARK9 [13], and VPS35 [14, 15].

Among these key players, mutations in the LRRK2 contribute to the most frequent cause of familial PD, and LRRK2 variants are also implicated to increase risk factors in the sporadic cases [16, 17]. Additionally, clinical features of LRRK2-associated PD patients are indistinguishable from the idiopathic cases, and most LRRK2 mutation carriers are positive for alpha-synuclein LBs [18]. Moreover, multiple lines of evidence support that LRRK2 interacts with other key molecules in the PD pathogenesis, including SNCA, Parkin, DJ-1 and PINK [19–21].

LRRK2 gene contains 51 exons and encodes a large 286kD complex protein of 2527 amino acids. It belongs to the ROCO family of proteins, characterized by a catalytic Ras of complex proteins (ROC) GTPase domain, a C-terminal of ROC (COR) domain and a kinase domain, which phosphorylates serine/threonine residues. This central tri-domain is flanked by various potential protein-protein interaction domains, including armadillo (ARM), ankyrin (ANK), leucine-rich and WD40 repeats [3, 22]. LRRK2 mRNA is widely expressed throughout the brain and other organs, including kidney, heart, lung, and liver [23]. In the brain, it is relatively high in the dopaminoceptive regions instead of dopaminergic neurons [24, 25].

LRRK2, also known as PARK8, was first discovered in autosomal-dominant, late-onset parkinsonism by genetic linkage analysis in 2002, and two years later, LRRK2 gene was cloned and its related mutations were reported [2, 3, 26]. Extensive works have been done to explore the pathophysiological role of LRRK2. Several pieces of data suggest that LRRK2 is involved in regulating neurite growth and cytoskeleton dynamics [27–29], maintaining functions of autophagy and lysosome [30–34], and modifying protein translation [35, 36] and vesicle trafficking [37–39]. There are over 75 substitutions have been found in LRRK2, and seven missense mutations (G2019S, I2020T, N1437H, R1441G/C/H and Y1699C) are pathogenic, all of which are concentrated in the central catalytic domains, suggesting an essential role of GTPase and kinase domains in the PD pathogenesis [40, 41].

Carboxyl terminus of HSP70-interacting protein (CHIP) was shown to interact with LRRK2 and be involved in regulating steady-state level of LRRK2 through ubiquitin proteasomal degradation pathway. Knockdown of CHIP was capable of exacerbating wildtype (WT) and mutant LRRK2-induced cell toxicity [42]. A more recent study implicated that the level of mutant LRRK2 was more predicative than kinase activity for its pathogenic effect and formation of inclusion bodies in neurons, suggesting manipulation of cellular level of LRRK2 is another option for treating LRRK2-associated PD [43]. Dysregulation of transcription was implicated in Alzheimer’s disease (AD), Huntington’s disease (HD) and PD [44–52]. A study found that LRRK2 mRNA was decreased in PD patients with comparison to control subjects [53]. However, it is still unknown that how LRRK2 is regulated at the transcriptional and translational level.

In this study we functionally analyzed human LRRK2 gene transcription. We first identified its transcription start site (TSS) and cloned its 1738 bp promoter region. There were multiple putative transcription factor-binding sites for various transcription factors, including Sp1, GATA1/2, *c-Jun*, HNF-3α, and NF-AT1. Furthermore, the transcription factor Sp1 was shown to promote human LRRK2 gene promoter activity and gene expression, whereas its inhibitor, mithramycin A (MTM), reduced the promoter activity and gene expression. This is the first study to examine the role of Sp1 signaling in regulating LRRK2 gene expression.

## Results

### Cloning the human LRRK2 gene promoter and mapping its transcriptional start site

**Fig. 1:**
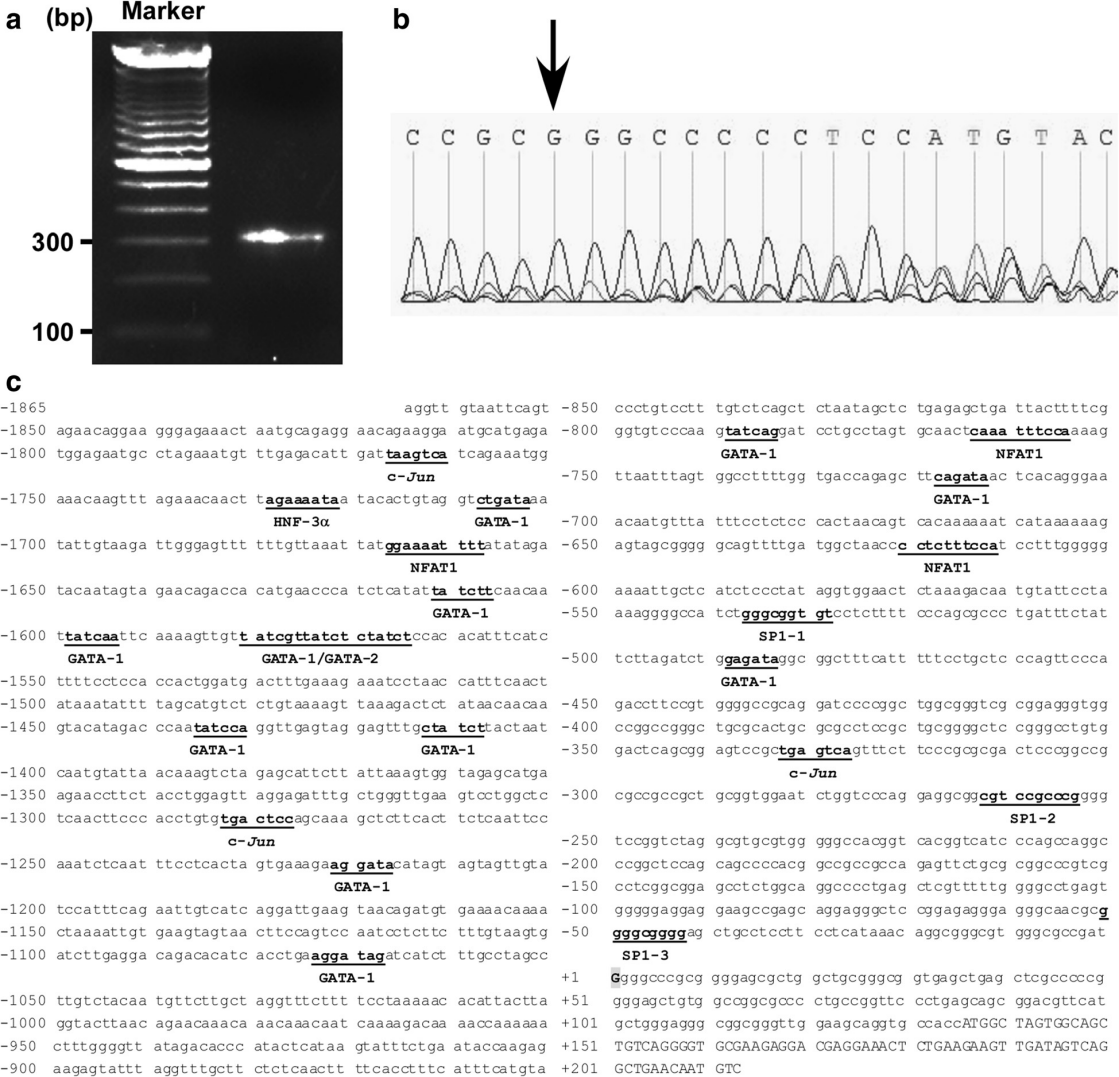
Identification of TSS and sequence features of the human LRRK2 gene promoter. **a** Smarter RACE cDNA amplification kit was used to amplify full-length cDNA from HEK293 cells. Nested PCR was performed and the product was resolved on 1.5 % agarose gel. **b** TSS was located by sequencing PCR product. The first base pair after SMARTer oligonucleotide is the TSS, which is indicated by arrow in Figure. **c** Sequence of the human LRRK2 promoter from -1865 bp to + 213 bp of the TSS (+1) is illustrated here. The putative transcription factor binding sites are underlined by computational search

To define the region of LRRK2 gene promoter, total RNA was extracted from HEK293 cell and 5’-rapid amplification of cDNA ends (RACE) assay was applied to identify the transcriptional start site of human LRRK2 gene. After amplifying the full length of LRRK2 cDNA, two pairs of primers were used to perform nested polymerase chain reaction (PCR). An ~300 bp band was yielded after inner PCR on 1.5 % agarose gel and the sequencing results indicated that the TSS was located 135 bp upstream of translational start site (ATG) (Fig. 1a, b). The transcriptional start site began with guanine and was designated as +1. To study the human LRRK2 gene promoter, a 1738 bp 5’-flanking region of human LRRK2 gene was cloned from HEK293 cell gDNA and the fragment was sequenced. A computational transcription factor search (PROMO, online tool) for 5’-flanking region of the human LRRK2 gene revealed that the human LRRK2 promoter contains several putative regulatory elements, including Sp1, GATA1/2, *c-Jun*, HNF-3α, and NF-AT1 (Fig. 1c).

### Functional analyses of the human LRRK2 gene promoter

**Fig. 2:**
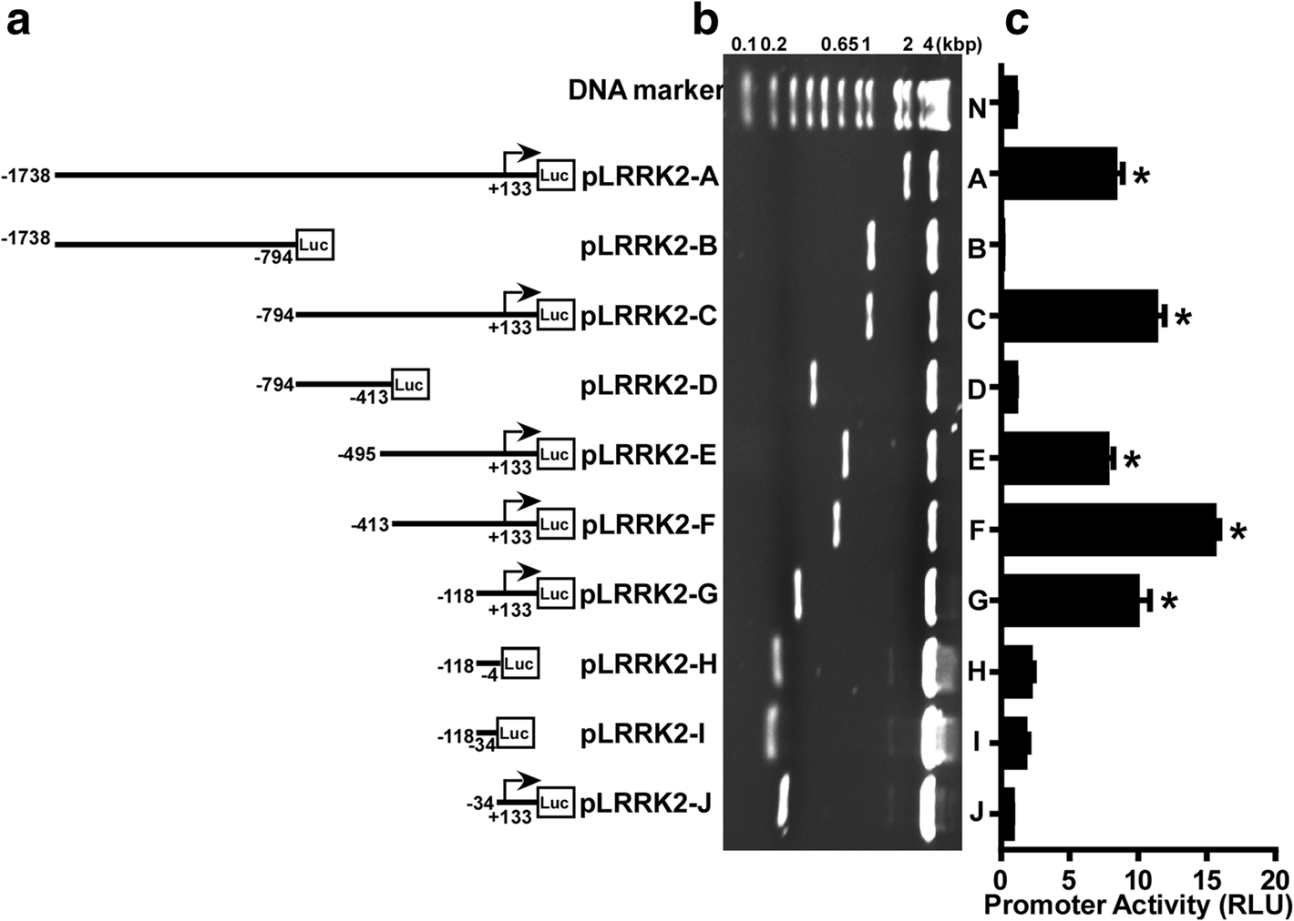
Functional deletion analyses of the human LRRK2 gene promoter. **a** Schematic illustration of human LRRK2 promoter constructs consisting a serial deletion fragments, which were cloned into pGL3-Basic plasmid. The arrows represent the direction of transcription and the numbers indicate the start and ending point of each construct with respect to TSS. **b** LRRK2 promoter constructs were verified by restriction enzyme digestion and the digested products were resolved on 1.5 % agarose gel. The size of vector is 4.8 kb and the size of inserts ranges from 84 to 1871 bp, which was further confirmed by sequencing. **c** Plasmids with different LRRK2 promoter constructs were cotransfected with pCMV-Luc into HEK293 cells. Cell lysates were harvested 24 h post-transfection, and the luciferase activity of pCMV-luc was used for normalizing transfection efficiency. The RLU of pGL3-Basic (marked as N) was designated as 1. The values represent means ± SEM, *n* =3, *p < 0.001, by analysis of variance (ANOVA) with Sidak’s multiple comparison test. Comparisons were made between all other columns and the pGL3-basic control column

To investigate the activity of human LRRK2 gene promoter, ten deletion fragments of its 5’-flanking region were cloned into pGL3-Basic vector (Fig. 2a). The construction of plasmids was verified by enzyme digestion (Fig. 2b). This vector lacks eukaryotic promoter and enhancer, but contains a *firefly* luciferase reporter gene. The expression of luciferase gene is driven by the correctly inserted promoter upstream of it. Constructed plasmids were transfected into cells and the inserted promoters’ activities were evaluated by the bioluminescent measurement of luciferase protein. The promoter activity of pLRRK2-A plasmid covering from −1738 bp to +133 bp was 8.27 ± 0.35 RLU, significantly higher than pGL3-Basic (*P* < 0.0001, Fig. 2c), indicating that this fragment worked as a functional promoter. To validate luciferase assay, four plasmids lacking TSS in its promoter region, including pLRRK2-B, pLRRK2-D, pLRRK2-H and pLRRK2-I, were served as experimental negative controls. As expectedly, all of four plasmids did not have promoter activities when comparing with pGL3-Basic (*p* > 0.05). A 944 bp deletion from pLRRK2-A to construct pLRRK2-C significantly increased promoter activities to 11.25 ± 0.38 RLU (*p* < 0.0001). Promoter activity of pLRRK2-E, containing a fragment −495 to +133 bp, was 7.70 ± 0.29 RLU, significantly weaker than pLRRK2-C (*p* < 0.0001). A further deletion from −495 bp (pLRRK2-E) to −413 bp (pLRRK2-F) drastically increased promoter activity to 15.52 ± 0.23 RLU (*p* < 0.0001) and a deletion of 295 bp from pLRRK2-F to create pLRRK2-G significantly lowered promoter activity to 9.89 ± 0.56 RLU (*p* < 0.0001). Importantly, when the fragment from −118 to −34 bp was deleted from pLRRK2-G to generate pLRRK2-J, promoter activity became negligible (0.78 ± 0.02 RLU). These data suggest that the fragment −118 to +133 bp has the minimum promoter activity required for transcription. Additionally, promoter regions from −1738 to −794 bp and −495 to −413 bp have negatively regulatory *cis*-acting elements and promoter region from −794 to −495 bp and −413 to −118 bp have upregulatory *cis*-acting elements.

### The human LRRK2 gene contains Sp1 binding sites

**Fig. 3:**
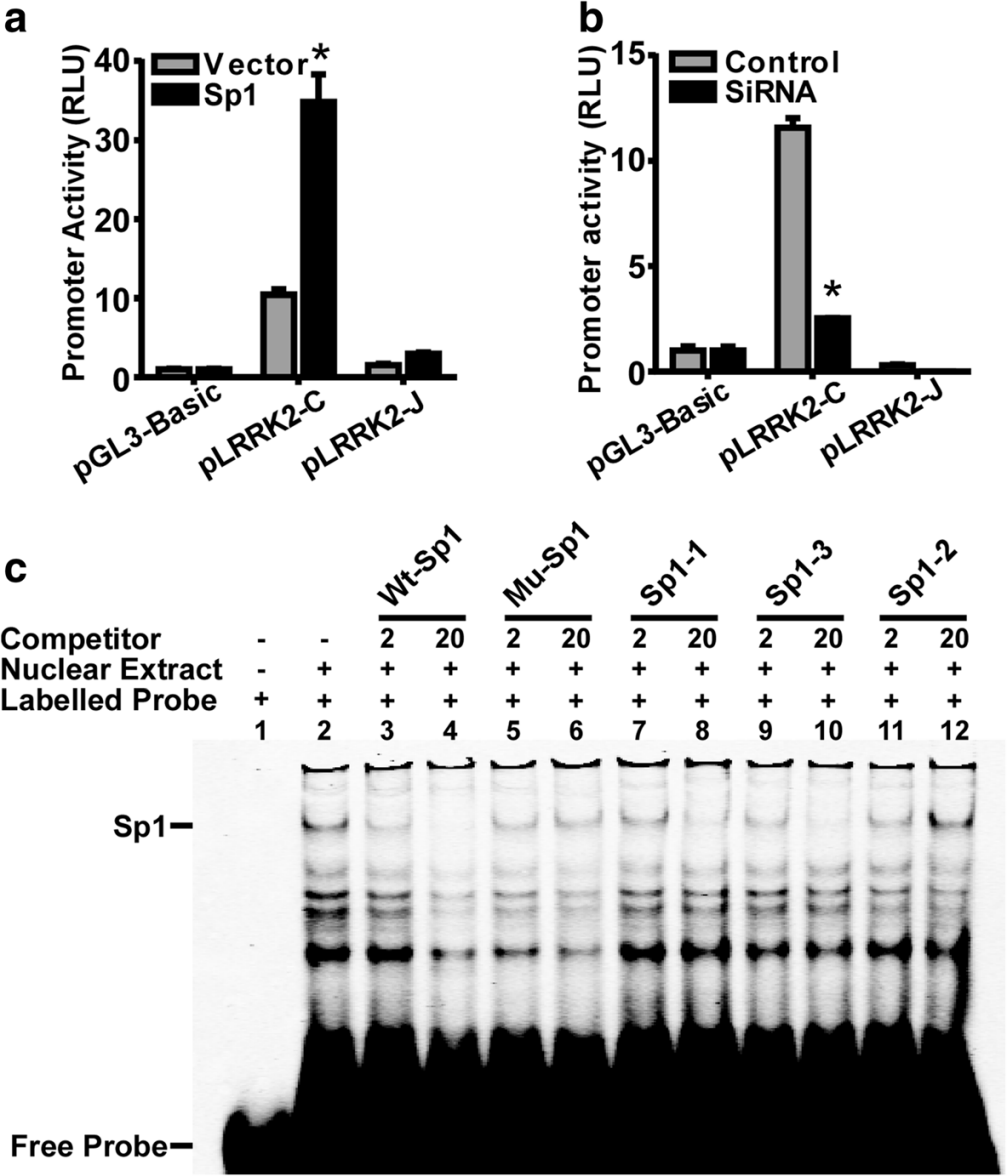
Regulation of the human LRRK2 gene promoter by Sp1. **a** pGL3-Basic, pLRRK2-C and pLRRK2-J plasmids were cotransfected with either vector or Sp1 expression plasmid into HEK293 cells. Cell harvesting and the measurement of luciferase activities were performed as mentioned before. Sp1 overexpression significantly increased the promoter activity of pLRRK2-C but had no effect on pLRRK2-J nor pGL3-basic control. The values represent means ± SEM. *n* =3, *p < 0.01 by two way ANOVA with Sidak’s multiple comparison test. **b** pGL3-Basic, pLRRK2-C and pLRRK2-J plasmids were cotransfected with either negative control or Sp1 siRNA into HEK293 cells. Knockdown of Sp1 significantly decreased the promoter activity of pLRRK2-C but had no effect on pLRRK2-J. The values represent means ± SEM. *n* =3, *p < 0.01 by two-way ANOVA with Sidak’s multiple comparison test. **c** EMSA was performed as described in detail in Material and Methods. Sp1 consensus binding sequence was labelled by fluorescent IR700 Dye. Lane1 is the labelled probe alone without nuclear protein extract. Incubation the probes with Sp1-enriched nuclear protein extracts formed a shifted DNA-protein complex band (lane 2). Competition assays were conducted by adding various concentrations of molar excess of unlabeled competitive oligonucleotides, including consensus Sp1 oligonucleotides (lane 3 and 4), mutant Sp1 consensus oligonucleotides (lane 5 and 6) and putative Sp1-responsive elements in the human LRRK2 promoter (lane 7 to 12)

**Fig. 4:**
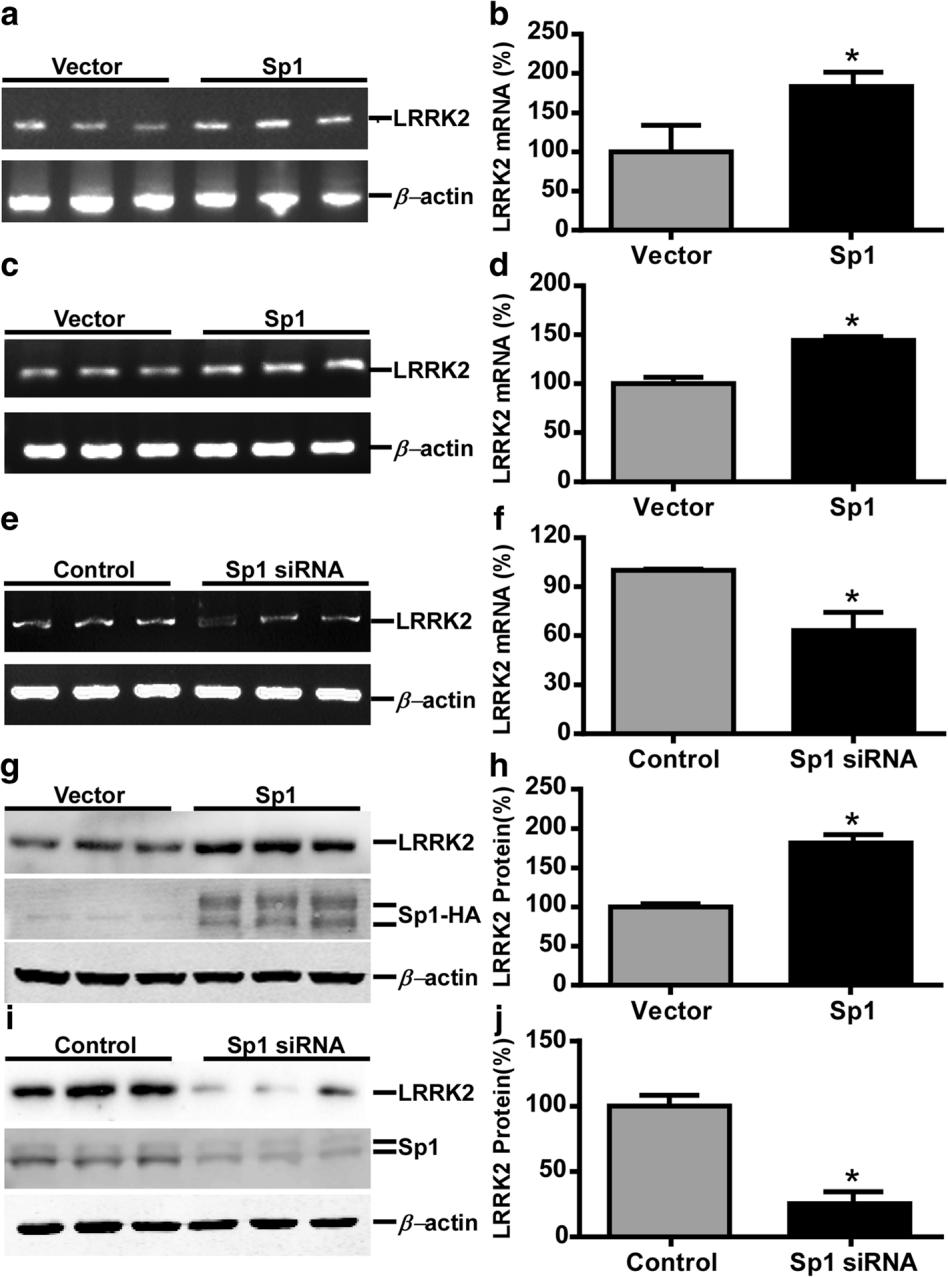
Sp1 upregulates the LRRK2 gene expression. **a-d** Sp1 overexpression increased LRRK2 mRNA expression level. Sp1 expression plasmid was transfected into HEK293 cells (**a**) or MN9D cells (**c**). Cell lysates were harvested 48 h after transfection and total RNA was isolated for RT-PCR. The products of amplified LRRK2 and β-actin genes were analyzed on a 1.5 % agarose gel. Quantification was performed by ImageJ software and endogenous LRRK2 mRNA level was normalized against β-actin. **e** HEK292 cells were transfected with scrambled siRNA or Sp1 siRNA, and endogenous LRRK2 mRNA level was measured by RT-PCR after 48 h and analyzed on a 1.5 % agarose gel. **g** HEK293 cells were transfected as mentioned before and cell lysates were harvested 48 h after transfection. Endogenous LRRK2 protein and overexpressed Sp1 were examined by immunoblotting. **i** HEK293 cells were transfected with negative control siRNA or Sp1 siRNA. After 48 h, cell lysate was harvested for determining LRRK2 and Sp1 protein level by immunoblotting. Quantification of the band intensity in (**f**), (**h**), and (**j**) was performed by ImageJ software. The values in this figure represent means ± SEM. *n* =3, *p < 0.05, analyzed by Student’s t-test

Computational transcription factor search (PROMO, online tool) the human LRRK2 gene revealed three putative sp1 binding sites in its promoter region, including −537 to −529 bp, −263 to −254 bp and −51 to −43 bp (Fig. 1c). To examine the effect of Sp1 on human LRRK2 gene promoter, the promoter activities of pLRRK2-C, which contained all three putative Sp1 binding sites, were measured in HEK293 cells cotransfected with Sp1 expression plasmids. The promoter activities of pLRRK2-J with no putative Sp1 binding sites were also examined to serve as a negative control (Fig. 3a). The results showed that the promoter activity of pLRRK2-C significantly increased from 10.43 ± 0.68 RLU to 34.79 ± 2.01 RLU after Sp1 overexpression (*p* < 0.001), but not for the promoter activity of pLRRK2-J (*p* > 0.05), demonstrating that Sp1 upregulated LRRK2 gene promoter activity in HEK293 cells. To confirm the specificity of Sp1’s effect on LRRK2 promoter activity, Sp1 siRNA was used to knock down all three isoforms for human Sp1, and a scrambled siRNA serves as a negative control. The endogenous Sp1 expression was significantly decreased in the HEK293 by the siRNA treatment cells (Fig. 4j). Knockdown of endogenous Sp1 significantly reduced the promoter activities of pLRRK2-C from 11.57 ± 0.46 RLU to 2.53 ± 0.01 RLU (*p* < 0.0001), but had no effect on plasmid pLRRK2-J (*p* > 0.05) (Fig. 3b).

To determine whether all three putative Sp1 binding sites were functional, EMSA was conducted to examine the binding between Sp1 and these promoter regions *in vitro* (Fig. 3c). Sp1 expression plasmid pCGN-Sp1 was transfected into HEK293 cells followed by extracting nuclear proteins from cell lysate. Double-stranded nucleotides containing the Sp1 consensus binding sequence (attcgatcgGGGCGGGgcgagc) were synthesized and labelled with IRD700 dye. Therefore, a free probe band can be observed on DNA polyacrylamide gel electrophoresis (PAGE) gel as shown in the first lane in Fig. 3c. A shifted band was visualized on DNA PAGE gel after adding Sp1- enriched nuclear extract (Fig. 3c, Lane 2). To ensure the specificity of this shifted band, WT oligonucleotides, containing Sp1 consensus binding sequence without labeling, and unlabeled mutant oligonucleotides were added to EMSA system. As expectedly, unlabeled WT oligonucleotides with 2- fold concentration of labelled probe successfully competed shifted band (Fig. 3c, Lane 3) and more excess of WT competitors further decreased intensity of the shifted band (Fig. 3c, Lane 4). On the contrary, mutant oligonucleotides with 2-fold and 20-fold concentration of labelled probe had little competing effect (Fig. 3c, Lane 5 and 6).

To test the functionality of three putative Sp1 binding sites located in the human LRRK2 gene promoter, double-stranded oligonucleotides were used to compete for the Sp1 consensus binding sequence. The oligonucleotides containing the first Sp1 binding site competed shifted band in a dosage-dependent manner (Fig. 3c, Lane 7 and 8). Similarly, the oligonucleotides containing the third Sp1 binding site also lowered the intensity of shifted band (Fig. 3c, Lane 9 and 10). However, the second Sp1 binding site did not show obvious competitive effect (Fig. 3c, Lane 11 and 12). Taken together, these data suggest that there are two functional Sp1 binding sites in human LRRK2 gene promoter and the binding between Sp1 and *cis*-acting elements on human LRRK2 promoter upregulated its promoter activities.

### Sp1 upregulates the LRRK2 gene expression

To determine whether Sp1 regulates LRRK2 gene expression, endogenous LRRK2 mRNA levels were measured after transfecting either pCGN-Sp1 expression plasmid or control vector into HEK293 cells. Sp1 overexpression resulted in a significant increase of LRRK2 mRNA level by 83.1 ± 18.4 % compared with control detected by reverse transcription (RT)-PCR (*p* < 0.05, Fig. 4a, b). Next, a dopaminergic cell line MN9D was used to confirm the effect. Similarly, the LRRK2 mRNA level was elevated to 144.10 ± 2.28 % with Sp1 overexpression in MN9D cells (*p* < 0.001, Fig. 4c, d). On the contrary, inhibition of endogenous Sp1 protein by siRNA led to significantly lower expression of LRRK2 mRNA in HEK293 cells (*p* < 0.01, Fig. 4e, f).

Although Sp1 drastically enhanced endogenous LRRK2 mRNA expression, it is not necessary that Sp1 can increase its protein level. Therefore, expression of endogenous LRRK2 protein was detected by immunoblotting after pCGN-Sp1 plasmid or control vector being transfected into HEK293 cells. In consistent with mRNA data, Sp1 significantly augmented LRRK2 protein level by 81.7 ± 6.21 % (*p* < 0.005, Fig. 4g, h). However, when Sp1 was overexpressed in MN9D cells, measurement of LRRK2 protein level was failed as endogenous LRRK2 protein cannot be detected in this cell line by any antibodies we tried (data not shown). Knockdown of endogenous Sp1 significantly lowered LRRK2 protein level by 74.52 ± 9.09 % (*p* < 0.005, Fig. 4i, j). Overall, the data suggested that Sp1 can not only promote LRRK2 gene promoter activity and also facilitate its gene expression both at transcriptional and translational level.

### Mithramycin A (MTM) inhibits human LRRK2 promoter activity and gene expression

**Fig. 5:**
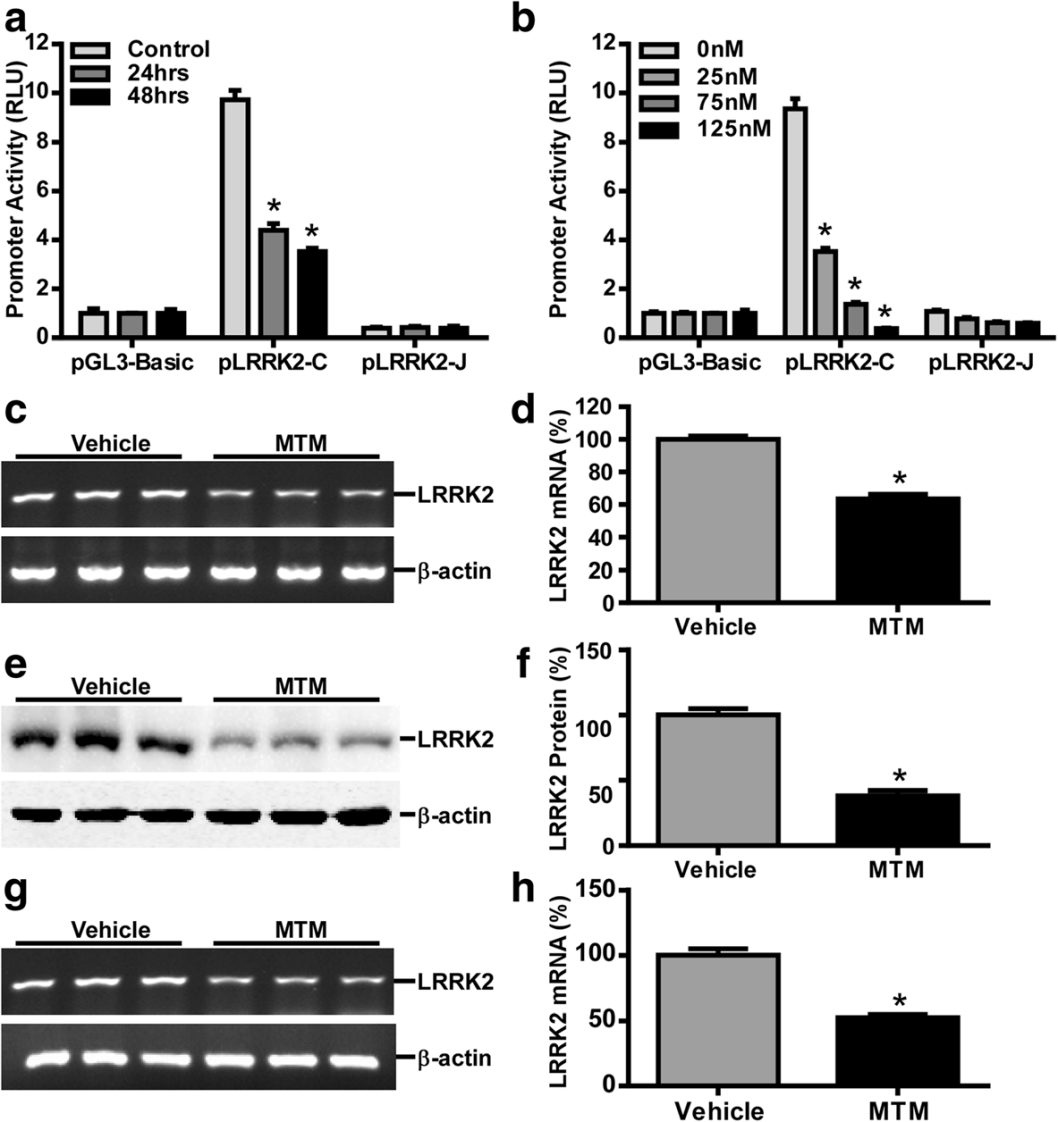
MTM inhibits the LRRK2 gene expression. **a** pGL3-Basic, pLRRK2-C or pLRRK2-J was transfected into HEK293 cells. After 24 h, transfected cells were treated with MTM at 125 nM or vehicle for 24 or 48 h. Luciferase activities were determined as mentioned before, and pCMV-Luc luciferase activity was used for transfection efficiency normalization. **b** HEK293 cells were transfected with pGL3-Basic, pLRRK2-C or pLRRK2-J. The next day, cells were exposed to MTM at 25, 75 and 125 nM for 24 h. Luciferase activities were measured. The values in (**a**) and (**b**) represent the mean ± SEM. *n* = 3, *p < 0.001 by two-way ANOVA with Sidak’s multiple comparison test. **c** HEK293 cells were treated with 125 nM MTM or vehicle for 24 h. The LRRK mRNA levels were determined by RT-PCR and normalized against the levels of β-actin. **d** Quantification of LRRK2 and β-actin mRNA levels in HEK293 cell were completed by ImageJ software. **e** Cell lysates harvested from HEK293 cells treated with 125 nM MTM or vehicle for 24 h were analyzed by immunoblotting with anti-LRRK2 antibody. β-actin was used as the internal control for protein loading. **f** Quantification of LRRK2 and β-actin protein levels in HEK293 cell was completed by ImageJ software. **g** Dopaminergic MN9D cells were treated with 125 nM MTM or vehicle for 24 h. The LRRK mRNA levels were determined by RT-PCR and normalized against the levels of β-actin. **h** LRRK2 and β-actin mRNA level in MN9D cell was quantified by ImageJ software. The values in (**d**), (**f**) and (**h**) represent the mean ± SEM. *n* = 3, *p < 0.001 by Student’s t-test

To further validate Sp1’s effect on LRRK2 gene expression, MTM, a selective Sp1 inhibitor competing with Sp1 to bind to a GC-rich DNA sequence [54], was used to treat cells. After HEK293 cells were transfected with pLRRK2-C or pLRRK2-J plasmids, MTM was applied to cells for either 24 or 48 h. With MTM treatment (125nM), the promoter activities of the pLRRK2-C plasmid, containing Sp1 binding sites, were significantly reduced from 9.72 ± 0.22 RLU to 4.40 ± 0.16 RLU after 24 h and further to 3.53 ± 0.08 RLU after 48 h (*p* < 0.0001 for both 24 and 48 h). Neither pLRRK2-J nor pGL3-Basic’s promoter activities was affected by MTM treatment (*p* > 0.05, Fig. 5a). To further confirm the effect of MTM treatment, various concentrations of MTM were applied to HEK293 cells, ranging from 25nM to 125nM. After 24 h treatment, promoter activities of pLRRK2-C plasmid were significantly downregulated from 9.35 ± 0.41 RLU to 3.53 ± 0.14 RLU in 25nM MTM treatment, further decreased to 1.37 ± 0.09 RLU in 75nM, and 0.38 ± 0.02 RLU in 125nM, respectively (*p* < 0.0001 for 25, 75 and 125nM. Fig. 5b). These data indicate that MTM treatment inhibits LRRK2 promoter activities in a time -dependent and dosage-dependent manner.

To examine LRRK2 mRNA expression in MTM treatment, HEK293 cells were administrated with 125nM MTM for 24 h. RT-PCR results showed that endogenous LRRK2 mRNA level was significantly downregulated to 63.43 ± 1.66 % (*p* < 0.0001, Fig. 5c, d). Endogenous LRRK2 protein expression was also detected in HEK293 cells after 125nM MTM treatment for 24 h. LRRK2 protein was reduced to 38.30 ± 2.18 % (*p* < 0.0001, Fig. 5e, f). LRRK2 mRNA expression after MTM treatment was confirmed in MN9D cells, which was significantly reduced to 52.44 ± 2.07 % (*p* < 0.001, Fig. 5g, h). Consistent with Sp1’s effect on LRRK2 gene, inhibition of Sp1’s activity by MTM is sufficient to reduce LRRK2 promoter activity and gene expression.

## Discussion

LRRK2 is one of the key players in the pathogenesis of PD, and its physiological and pathophysiological functions are studied extensively. LRRK2 is widely expressed with relative low expression in dopamine-producing area [24]. LRRK2 mRNA was found to be increased in the PD subjects and a study suggests that the level of mutant LRRK2 is associated with its toxic effect in neurons [43, 53]. However, the transcriptional and translational regulation of LRRK2 gene is still not known. In this study, we cloned and functionally characterized LRRK2 promoter. The transcription stat site of the human LRRK2 promoter was identified and the minimal promoter required for transcriptional initiation was located. Two Sp1-responsive elements were mapped in its promoter region and Sp1 was capable of promoting endogenous LRRK2 mRNA and protein expression in the cellular models. Contrarily, application of MTM reduced LRRK2 gene expression.

LRRK2 is a complex protein, featuring a central ROC-COR domain and a kinase domain. Its cellular functions were revealed by various loss-of-function studies. It was showed that deletion of LRRK2 in primary neurons resulted in longer neurites and elevated branching, the mechanisms of which were involved decreased phosphorylation of ezrin, radixin, and moesin (ERM) and filamentous actin [27, 28]. Deficiency of LRK1, homolog of the human LRRK2 in *C. elegans,* led to the disturbance of polarized location of synapses, suggesting that LRRK2 plays a role in vesicle trafficking [55]. Additional data emerging from LRRK2 knockout mice indicated that they actually did not severely compromise dopaminergic functions, including normal dopaminergic synthesis, release and storage [56]. However, the deficit was obvious in kidney with striking degeneration. This observation was confirmed by another study, and the abnormalities in the study were also seen in lung, both of which was caused by impaired autophagy-lysosomal pathway [34].

As most of the pathogenic mutations are concentrated in the central tri-domain, LRRK2’s kinase and GTPase activities have garnered significant attention [41]. Overexpression of mutant LRRK2 resulted in cell death and inclusion formation in neuronal cells and primary neurons, and increased kinase activities were linked to the underlying mechanisms, especially for LRRK2 G2019S mutant with consistently findings of its elevated kinase activities [19, 57–59]. LRRK2 mutants with reduced kinase activities were correlated with decreased neuronal toxicities and kinase-dead versions blocked inclusion formation and attenuated cell death [57, 60]. However, it is challenging to conclude that kinase activity is the major culprit of cell toxicity, as kinase activities of other LRRK2 mutants are controversial, sometimes no influence, and sometimes decreased [61]. Another candidate for pathogenic effect of LRRK2 mutations is the GTPase ROC domain. It is well established that LRRK is an authentic GTPase and PD-associated mutations located in the ROC domain (R1441G/C/H) and COR domain (Y1699C) decrease the rate of GTP hydrolysis [62–66]. Multiple lines of evidence supported that both GTP binding and GTP hydrolysis were required for kinase activity, but kinase-dead mutant did not have impact on GTP binding [67, 68]. Expression of GTPase domain alone was sufficient to impair yeasts’ viability, but the fragment only containing kinase domain produced much less toxic effect. Moreover, the fragment containing the complete central tri-domain was the most toxic one, suggesting kinase domain may have a modulating effect on GTPase activity [69]. Collectively, it remains unclear that whether kinase activity or GTPase activity is the readout of LRRK2’s pathogenic effect, but it is certain that both of them play an essential role in regulating the overall function of LRRK2.

Although LRRK2 has become a hot topic in the field of PD-related studies, the features of human LRRK2 promoter has not been studied in detail. A previous study suggested that there were at least six TSSs, ranging from 48 to 120 bp upstream of the first Kozak sequence, for LRRK2 promoter by using human brain cDNA [58]. Our identified TSS was 15 bp further, possibly due to cell-type or tissue-type specific effect [70]. In the human LRRK2 promoter sequence we cloned, there were multiple putative binding sites for various transcription factors including Sp1, *c-Jun*, HNF-3α, GATA-1/2, and NFAT1. Dysregulation of Sp1 was reported in various neurodegenerative disorders. Sp1 mRNA and protein level were increased in frontal cortex of AD brains, and the same results were also found in frontal cortex and hippocampus of AD model mouse [71]. It was discovered that huntingtin interacted with Sp1 and its coactivator, TAFII130, by using the yeast two-hybrid assay, and the interaction between Sp1 and TAFII130 was inhibited by mutant huntingtin in HD subjects [44]. Additionally, our lab determined that Sp1 was able to regulate several AD- and HD- associated genes, including BACE1, huntingtin and SNAP-25 [48, 72, 73].

Sp1 was one of the first transcription factors to be cloned in the 1980s [74]. It was originally discovered as a transcription activator for simian virus 40 (SV40) by binding to multiple GC-boxes in its early promoter [75]. Sp1 is ubiquitously expressed and involved in cell growth, cell differentiation, embryogenesis, and preventing CpG islands from methylation [76–78]. There are four major domains (A, B, C and D), featuring by two classic zinc fingers located in domain C for sequence-specific DNA binding and domain A and B at the N-terminal for transcription activation [79, 80]. As a classic transcription factor, Sp1 can bind to GC-box and GT/CACCC-box [81, 82]. Its consensus binding sequence is (G/T)GGGCGG(G/A)(G/A)(C/T) [82]. Our data show that in human LRRK2 promoter, the first putative binding site has sequence of GGGCGGTGC, the second CGTCCGCCCG, and the third GGGGCGGGGA. The first putative binding site has two mismatched base pairs, the second with three mismatched base pairs, and the third with only one mismatched base pair. In consistent with prediction, only the first and the third were functional binding sites.

MTM, an anti-tumor and antibiotic drug, was discovered to bind to GC-rich sequence with high affinity [83]. It competitively binds to Sp1 consensus binding site on the SV40 promoter, working as a site-specific inhibitor for Sp1 [84]. As expectedly, application of MTM resulted in significantly reduced LRRK2 promoter activity and gene expression. It was reported that LRRK2 WT or G2019S transgenic mice alone did not develop neuropathological features seen in the SNCA A53T transgenic mice, including astrocytosis, microgliosis, and neurodegeneration. However, SNCA A53T/LRRK2 WT double transgenic mice displayed increased reactive astrocytosis, microgliosis and neuronal death. Furthermore, the severity of neurodegeneration in the double transgenic mice was associated with expression level of WT LRRK2 proteins, suggesting an essential role of LRRK2 expression level in promoting mutant SNCA-induced neuropathology [85]. Therefore, decreasing the LRRK2 level by MTM could be beneficial to alleviate the PD-related pathological alterations, although the off-target effects of Sp1 inhibition should be taken into consideration. Further studies will be necessary to explore the effect of manipulating Sp1 signaling, for example by application of MTM, on LRRK2 WT or mutant-induced toxicity in PD model mice.

## Conclusions

In summary, we functionally analyzed human LRRK2 gene transcription, identified its TSS and cloned its 1738 bp promoter region. Furthermore, the transcription factor Sp1 was shown to promote human LRRK2 gene promoter activity and gene expression, whereas its inhibitor, MTM, reduced the promoter activity and gene expression. This is the first study to demonstrate that Sp1 signaling plays an important role in regulating LRRK2 gene expression.

## Methods

### Cloning and plasmids

LRRK2 gene promoter fragments were amplified from genomic DNA of human embryonic kidney (HEK) 293 cells by PCR and then cloned into pGL3-Basic expression vector (Promega) upstream of the luciferase reporter gene by restriction enzymes. Ten promoter deletion plasmids for 5’ flanking region of human LRRK2 gene were generated to cover from −1738 bp upstream to +133 bp downstream of TSS at guanine (+1). The primers, including restriction enzyme sites, were synthesized as follows: forward, 1) - 1738NheI: ctagctagcgaaacaacttagaaaataatacactg, 2) -794NheI: ctagctagccccaagtatcaggatcctgcc, 3) -495 BglII: cttagatctggagataggcggc, 4) -413Nhe: ctagctagcggtcgcggagggtggccggc, 5) -118XhoI: ccgctcgagtcgtttttgggcctgagt, and 6) -34XhoI: ccgctcgagtccttcctcataaacaggcg; reverse, 1) -794HindIII: cccaagcttggcaggatcctgatacttggg, 2) -413HindIII: cccaagcttgccggccaccctccgcgacc, 3) -34HindIII: cccaagcttaggcagctccccgccccgcgt, 4) -4HindIII: cccaagcttgcgcccacgcccgcctgttta, and 5) +133HindIII: cccaagctttggcacctgcttccaacccgccg.

### Cell culture, transfection, luciferase reporter assay and MTM treatment

HEK293 cells and MN9D cells were cultured in Dulbecco’s modified Eagle’s medium (DMEM) supplemented with 10 % fetal bovine serum, 1 mM of sodium pyruvate, 2 mM of L-glutamine, 50 units of penicillin and 50 μg of streptomycin (Invitrogen). MN9D cells were cultured on the plates coated with 10 μg/mL poly-D-lysine (Sigma). All cells were maintained at 37 °C in an incubator containing 5 % CO_2_. Sp1 expression plasmid was constructed by inserting Sp1 cDNA with hemagglutinin (HA) tag into pCGN-expression plasmid under the control of the cytomegalovirus promoter [86]. Transfection for overexpressing Sp1 in the HEK293 and MN9D cells were performed with Lipofectamine 2000 (Invitrogen) following manufacturer’s instruction.

For luciferase assays, cells were first cotransfected with 500 ng *firefly* luciferase plasmid (pGL3-Basic) with insertion of various promoter fragments and 1 ng *Renilla* luciferase plasmid pCMV-Luc which was used to normalize for transfection efficiency. Cells were harvested by passive lysis buffer 24 h post-transfection. *Firefly* luciferase and *Renilla* luciferase activities were measured by using the dual-luciferase reporter assay system (Promega). The *firefly* luciferase activities were normalized to *Renilla* luciferase activities and the promoter activities of various deletion fragments were represented as relative luciferase units (RLU) after normalizing to pGL3-Basic.

MTM (Sigma) was dissolved in 100 % methanol to make a stock concentration of 250 mM. In dosage experiments, HEK293 cells were treated with MTM at 0, 25, 75 and 125 nM for 24 h after 1 day transfection. Similarly, for time course experiments, cells were treated with MTM at 125 nM for 24 or 48 h. For RT-PCR and immunoblotting, HEK293 and MN9D cells were treated with 125 nM MTM for 24 h and then lysed for mRNA and protein extraction.

### 5’- RACE assay

Total RNA was extracted from HEK293 cells by TRI reagent following the manufacturer’s instructions (Sigma). 5’- RACE was conducted using the Smarter RACE cDNA amplification kit (Clotech) according to its protocol. In the reverse transcription, a patent SMARTScribe Reverse Transcriptase was employed to generate full-length first-strand cDNA and 3-5 bp residues were added to its 3’ tailing. The SMARTer oligonucleotide was annealed to the extended cDNA tail, and the oligonucleotide was then worked as a template to amplify a complete cDNA copy of the original RNA with the additional SMARTer sequence at the end. The outer and inner reverse primers were designed based on human LRRK2 gene sequence, which were 5’-atcccagccatcatccagacc and 5’- caggatttggaccagcgtttct, respectively. Nested PCR was performed and PCR product was sequenced to locate the TSS of the human LRRK2 gene, which was the first base pair after SMARTer oligonucleotide sequence.

### EMSA

EMSA was performed as previously described [87]. HEK293 cells were transfected with pCGN-Sp1 expression plasmid and lysed in a series of hypotonic buffers for nuclear protein extraction. Probe oligonucleotides were labelled with IR700 Dye (LI-COR Biosciences) and annealed to produce double- stranded probes. The labelled probes were incubated with or without nuclear extract at 22 °C for 20 min in the EMSA binding buffer (4 % glycerol, 1 mM MgCl_2_, 0.5 mM EDTA, 0.5 mM DTT, 50 mM NaCl, 10 mM Tris-HCl (pH 7.4), and 50 μg/mL poly(dI-dC)). For the competition assay, nuclear extract was first incubated with 100 fmol (2 times excess) or 10 pmol (20 times excess) of unlabeled competition oligonucleotides for 10 min followed by adding 50 fmol labelled probes. The sequences of the oligonucleotides were: consensus Sp1-forward: 5’-attcgatcggggcggggcgagc; consensus Sp1-reverse: 5’-gctcgccccgccccgatcgaat; mutant Sp-1 forward: 5’-cccttggtgggttgggggcctaagctgcg; mutant Sp-1 reverse: 5’-cgcagcttaggcccccaacccaccaaggg ; LRRK2-Sp1-PROBE1-forward: 5’-gccatctgggcggtgtcctc; LRRK2-Sp1-PROBE1-reverse: 5’gaggacaccgcccagatggc; LRRK2-Sp1-PROBE2-forward: 5’-gcggcgtccgcccggggtcc; LRRK2-Sp1-PROBE2-reverse: 5’-ggaccccgggcggacgccgc; LRRK2-SP1-PROBE3-forward: 5’-caacgcggggcggggagctg; LRRK2-Sp1-PROBE3-reverse: 5’-cagctccccgccccgcgttg. The EMSA samples were analyzed on 4 % non-denaturing polyacrylamide gels and the gels were scanned using the Odyssey scanner (LI-COR Biosciences) at a wavelength of 700 nm.

### Sp1 knockdown

HEK293 cells were maintained at 30 % confluence for transfection. For luciferase assay, the cells were cotransfected either 50nM Silencer® Select negative control siRNA or Sp1 siRNA (Thermofisher) with other promoter plasmids by Lipofectamine 2000 (Invitrogen) following manufacturer’s instruction. Cells were analyzed 48 h after transfection. For RT-PCR and immunoblotting, 50nM negative control siRNA or Sp1siRNA was transfected into HEK293 cells by Lipofectamine 2000. Cells were harvested 48 h after transfection. The sense sequence of Sp1 siRNA is 5’-gcaacaugggaauuaugaatt and the antisense sequence is 5’-uucauaauucccauguugctg.

### RT-PCR

Total RNA was extracted from HEK293 or MN9D cells by TRI reagent (Sigma). Thermoscript™ RT-PCR system (Invitrogen) was applied to amplify the first strand cDNA by using 1.0 μg of total RNA as the template and then the newly synthesized cDNA was further amplified by Taq DNA polymerase. The specific primers for human LRRK2 gene were as follows: forward, 5’- gagcacgcctccaagttat, and reverse, 5’- gtgattttacctgaagttag. This pair of primers was used to amplify a 302 bp fragment of the human LRRK2 gene coding sequence in the HEK293 cells. Additionally, the pair of primers for amplifying a 115 bp fragment of mouse LRRK2 gene coding sequence in MN9D cells was as follows: forward, 5’- aggagctgcccccttgaagaca, and reverse, 5’- tgtgccacaccctccccatgt. β-actin was used as an internal control, and two pairs of gene specific primers for HEK293 and MN9D cells were: forward, 5’- ggacttcgagcaagagatgg, reverse, 5’-gaagcatttgcggtggag, forward, 5’-gacaggatgcagaaggagat, and reverse, 5’-ttgctgatccacatctgctg, respectively. All samples were analyzed on 1.5 % agarose gels.

### Immunoblotting

HEK293 and MN9D cells were lysed in triton lysis buffer (150 mM sodium chloride, 1.0 % Triton X-100, 50 mM Tris-HCl (pH 8.0) and protease inhibitor cocktail (Roche), followed by brief sonication. Protein concentration was measured by Bradford assay (Bio-rad) and 4x sodium dodecyl sulfate (SDS) sample buffer was added to each sample. Cell lysates were resolved by 6 % Tris-glycine SDS-PAGE for detecting LRRK2 and 8 % Tris-glycine SDS-PAGE was used to detect endogenous Sp1 and Sp1-HA. Rabbit anti-LRRK2 monoclonal antibody MJFF C81-8 (Abcam), rabbit anti-Sp1 polyclonal antibody PEP2 (Santa Cruz), mouse anti-β-actin monoclonal antibody AC-15 (Sigma) and mouse anti-HA monoclonal antibody 12CA5 (Abcam) were used as primary antibodies. IRDye 680RD-labelled goat anti-rabbit antibodies and IRDye 800CW-labelled goat anti-mouse antibodies were applied as secondary antibodies. The gels were scanned in the Odyssey system (LI-COR Biosciences).
